# The PainChek® Pain Assessment Tool: Harnessing AI and Reducing Subjectivity to Assess Pain in People with Dementia

**DOI:** 10.1093/geroni/igaf122.2928

**Published:** 2025-12-31

**Authors:** Wingyun Mak, Orah Burack, Kreshnik Hoti, Jeff Hughes, Kimberly Bergen-Jackson

**Affiliations:** The New Jewish Home Research Institute on Aging, New York, New York, United States; The New Jewish Home, New York, New York, United States; PainChek, Sydney, New South Wales, Australia; PainChek, Sydney, New South Wales, Australia; University of Iowa, Iowa City, Iowa, United States

## Abstract

Pain assessment in people with dementia is challenging and can result in inadequate treatment. Most pain assessment tools require subjective quantification of pain/discomfort. PainChek® is a new assessment tool that harnesses a) artificial intelligence to provide objective pain ratings of facial expression and b) dichotomous ratings of observable non-facial pain indicators. This study aimed to validate PainChek® by examining the agreement, reliability, and predictive validity between PainChek® and the Abbey Pain Scale (APS), a more subjective pain scale commonly used in Australia. Participants (N = 103) were US nursing home residents with moderate-to-severe cognitive impairment. Participants were assessed for pain during rest and post-movement by two blinded raters, each administering one pain assessment tool. Agreement between raters was 92.7% (no pain), 69.1% (mild), 75% (moderate), and 83.3% (severe), suggesting high levels of agreement between PainChek® and APS assessments. Agreement rates, except in the mild condition, exceeded a priori hypotheses and were similar across rest/post-movement conditions. Intraclass correlations show that PainChek® (.73, 95% CI: .62-.81) outperformed APS (.59, 95% CI: .44-.71) on test-retest reliability. Bootstrapping methods yielded predictive values that were 75.5%, 89%, 82.8%, and 38.3% for no pain, mild, moderate, and severe. Low predictive value for severe pain was likely due to few occurrences of severe pain assessments. Results suggest that PainChek® performs on par with the APS, while providing a more objective format that enables greater accessibility for a variety of staff to administer reliably. Decreasing subjectivity from the pain assessment process may facilitate accuracy and lead to more appropriate treatment.

